# Metronomic chemotherapy in cancer treatment: new wine in an old bottle

**DOI:** 10.7150/thno.95619

**Published:** 2024-06-01

**Authors:** Huai-liang Wu, Han-xing Zhou, Li-min Chen, Shu-sen Wang

**Affiliations:** State Key Laboratory of Oncology in South China, Guangdong Provincial Clinical Research Center for Cancer, Sun Yat-sen University Cancer Center, 651 Dongfeng Road East, Guangzhou 510060, China.

## Abstract

Over the past two decades, metronomic chemotherapy has gained considerable attention and has demonstrated remarkable success in the treatment of cancer. Through chronic administration and low-dose regimens, metronomic chemotherapy is associated with fewer adverse events but still effectively induces disease control. The identification of its antiangiogenic properties, direct impact on cancer cells, immunomodulatory effects on the tumour microenvironment, and metabolic reprogramming ability has established the intrinsic multitargeted nature of this therapeutic approach. Recently, the utilization of metronomic chemotherapy has evolved from salvage treatment for metastatic disease to adjuvant maintenance therapy for high-risk cancer patients, which has been prompted by the success of several substantial phase III trials. In this review, we delve into the mechanisms underlying the antitumour effects of metronomic chemotherapy and provide insights into potential combinations with other therapies for the treatment of various malignancies. Additionally, we discuss health-economic advantages and candidates for the utilization of this treatment option.

## Introduction

In the continuously evolving field of modern oncology, a diverse range of novel treatment modalities are capturing the attention of clinical scientists. Notably, targeted therapy, immunotherapy, hormonal therapy, and other advanced treatment modalities have achieved remarkable success 1-5, have profoundly reshaped the therapeutic anticancer paradigm and have seemingly reduced the prominence of chemotherapy as the predominant systemic approach to cancer treatment. Nevertheless, these emerging anticancer therapies, in numerous completed and ongoing clinical trials, continue to rely on combination regimens with chemotherapy, potentially underestimating that chemotherapy alone may affect patient health in positive and negative ways based on dose, schedule, and mechanism of action 6-8. Conventional chemotherapy follows the maximum tolerated dose (MTD) paradigm and is associated with significant adverse effects, such as high systemic toxicity 9, 10. This is especially true if the drug is not targeted, which leads to patient deterioration and delayed tumour growth, as resistance to further chemotherapeutic dosages is established 11. However, metronomic chemotherapy (MCT) emphasizes the administration of low-dose and more frequent delivery of cytotoxic drugs to elicit a prolonged anticancer effect while concurrently minimizing toxicity 12, 13.

Over the past two decades, the implementation of MCT has not only demonstrated favourable outcomes in various randomized clinical trials within palliative settings 14, 15 but has also yielded progression-free survival benefits in patients with metastatic solid cancers when used either as a maintenance treatment or in combination with other therapies 16-19. Recently, the scope of MCT indications has been further extended as an adjuvant treatment for high-risk patients with locoregionally advanced breast cancer and nasopharyngeal carcinoma 20, 21. Additionally, numerous studies have revealed additional mechanisms of action of MCT, including its impact on the regulation of the immune tumour microenvironment (TME), direct tumour cell death and metabolic reprogramming, which has broadened our understanding of the antitumour activity of MCT beyond its originally perceived antiangiogenic mechanisms 22-24. This review presents an overview of the advancements in MCT and its mechanisms in cancer treatment. Furthermore, we summarize the clinical significance of MCT and its synergistic effects with other therapies. In the era of precision medicine, we want to emphasize that the “old bottles” of “MCT” regimens are constantly being filled with “new wine” and should be put in front of the “older bottles” of MTD already on the shelf.

## Evolving concept of MCT

**Figure 1:**
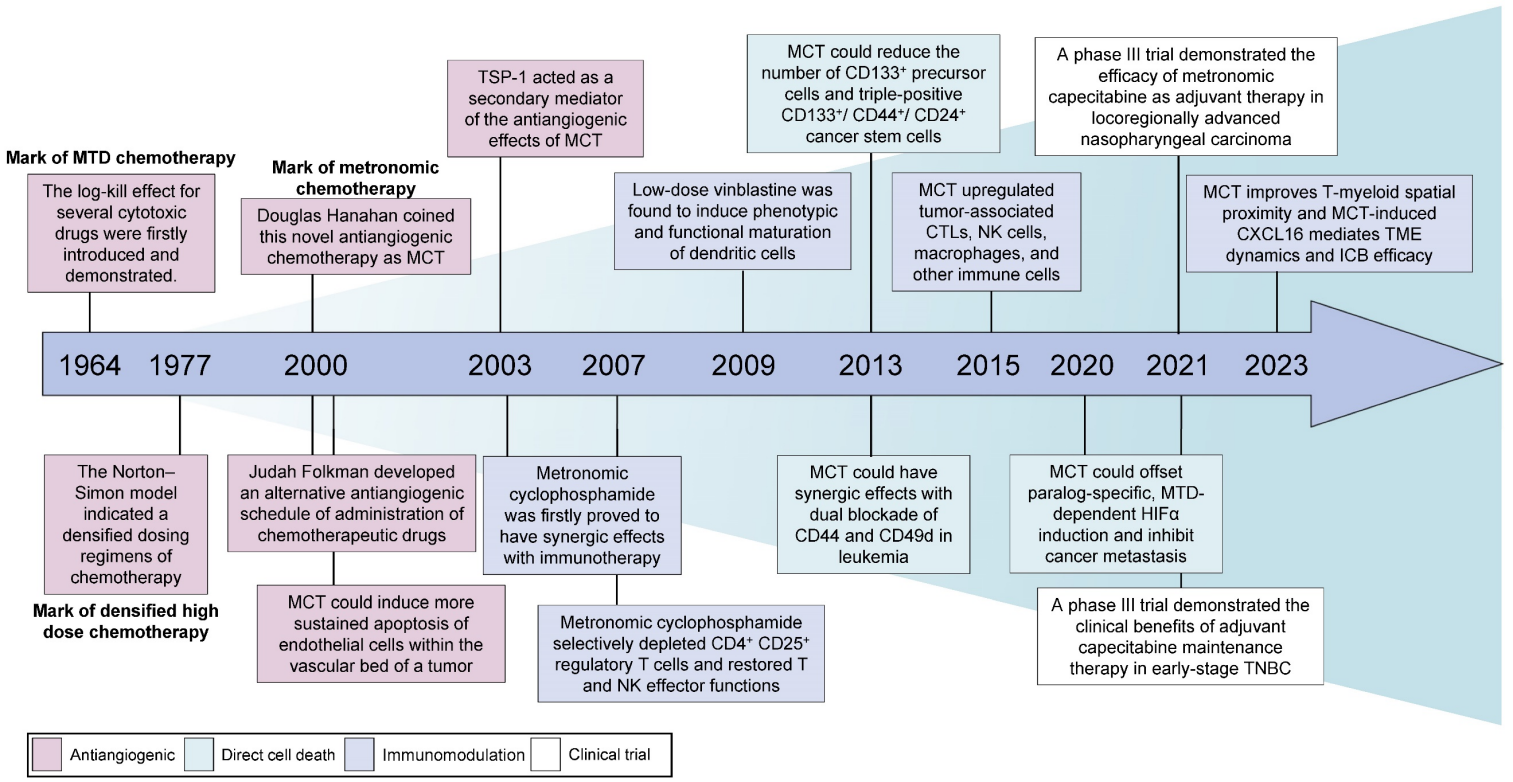
** Historical development and breakthroughs of MCT in cancer treatment.** MCT: Metronomic chemotherapy; MTD: Maximum tolerated dose; CTLs: Cytotoxic T lymphocytes; NK: Natural killer; TME: Tumour microenvironment; ICB: Immune checkpoint blocker; HIF-1α: Hypoxia-inducible factor 1; TNBC: Triple-negative breast cancer.

Sixty years ago, Skipper, Schabel and Wilcox were the first to introduce theoretical concepts for the optimal design of chemotherapies based on the log-kill effect of several cytotoxic drugs (Figure 1) 25. Additionally, their findings indicated that a large-dose/short-time schedule yielded better results than a frequent low-dose schedule with a similar total dose 26. Consequently, conventional cytotoxic drugs were administered in single doses or as short courses of therapy at the highest possible doses that did not result in life-threatening levels of toxicity 27. This approach, known as the 'maximum tolerated dose', gained prominence and became the dominant chemotherapy schedule. In the 1970s, Norton and Simon re-examined the Skipper-Schabel-Wilcox log-kill hypothesis and proposed that these chemotherapeutic drugs exhibit activity specifically against actively proliferating cells (Figure 1) 28. Moreover, the log-kill effect theory fails to explain the limited antitumour effects of chemotherapy on both small tumours and very large tumours 9. Consequently, the Norton-Simon model was developed to address this limitation, which led to the implementation of densified high-dose chemotherapy schedules in clinical practice 29, 30.

During the initial phases of chemotherapy, physicians suggested shifting the therapeutic goal from complete eradication of the tumour to long-term management of the disease 31. Several clinical precedents aimed for better disease control and revealed the potential antitumour effectiveness of MCT. Kakolyris *et al.* observed that a subset of patients with non-small cell lung cancer (NSCLC), metastatic breast cancer or ovarian cancer exhibited resistance to conventional chemotherapy but showed positive responses when the same drugs were administered orally at low doses but at a relatively high frequency 32. This novel approach, which involves the frequent administration of low-dose cytotoxic drugs, emerged in the year 2000 as a departure from the conventional MTD chemotherapy paradigm 33, 34. In 2000, Judah Folkman and Timothy Browder developed an alternative antiangiogenic schedule for the administration of cyclophosphamide 33. Douglas Hanahan described the concept of this less toxic and continuous chemotherapy and originally coined the term 'MCT' 34, 35. Although no universal definition of MCT has been accepted, MCT is defined as the minimum biologically effective dose of a chemotherapeutic agent, which still induces antitumour activity, given as a continuous dosing regimen without prolonged drug-free breaks 36. Other alternative terminologies of MCT include low-dose antiangiogenic chemotherapy, low-dose maintenance chemotherapy and metronomic scheduling of chemotherapy 13.

MCT was initially utilized as a single-drug treatment 37, 38. However, as a deeper understanding of its mechanisms has emerged, clinicians have begun to frequently combine MCT with nonchemotherapeutic drugs, such as antiangiogenic drugs and targeted therapies 39-41. Although MCT was originally defined as an antiangiogenic anticancer strategy 27, 42, additional mechanisms, including immunomodulation, elimination of cancer stem cells, and metabolic reprogramming, have since been revealed 43-45.

## Mechanisms and rationale of MCT

### Antiangiogenic effects on endothelial cells

**Figure 2:**
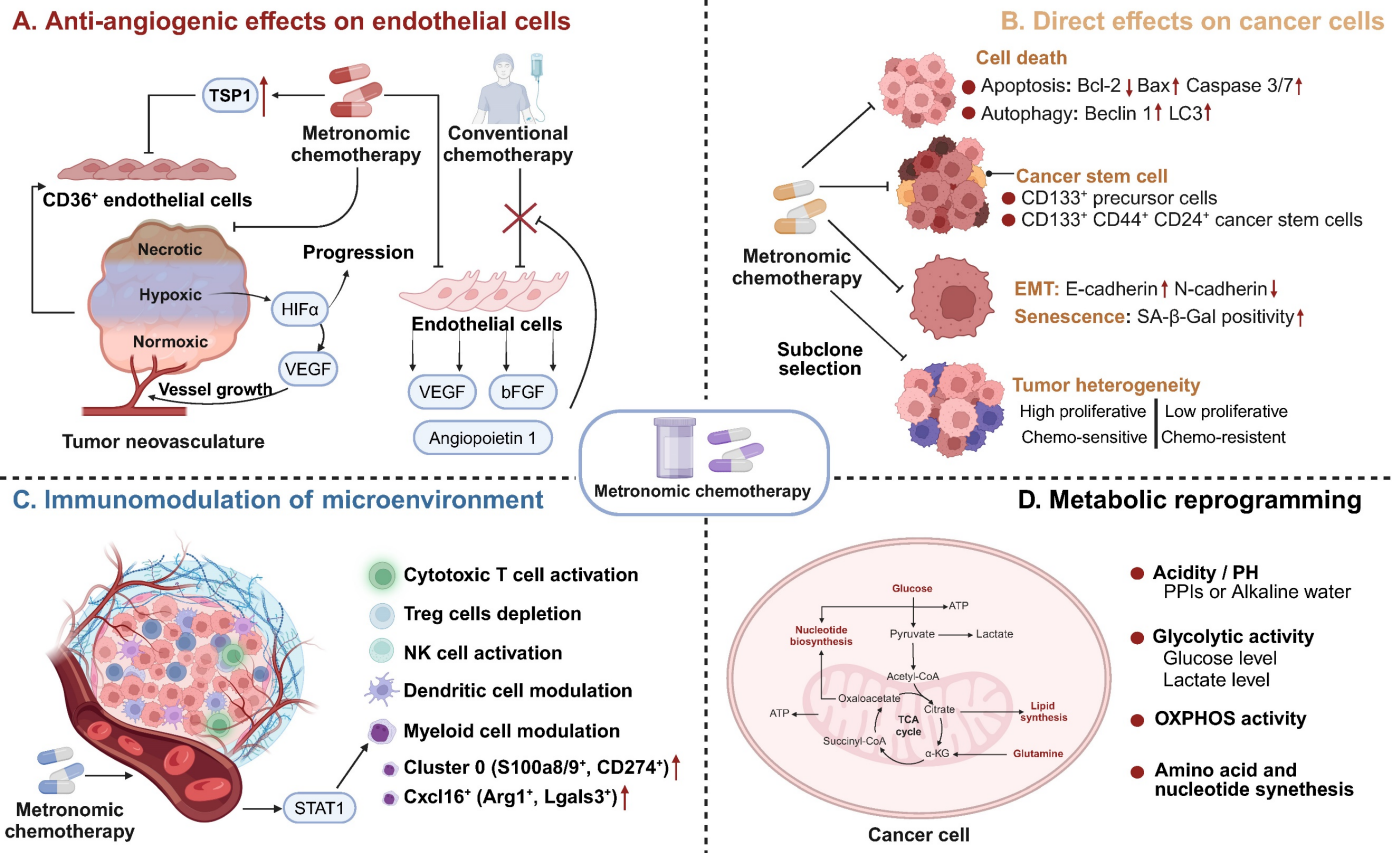
** Mechanisms of Action of MCT.** MCT could induce antitumour effects via **A** Antiangiogenic effects on endothelial cells; **B** Direct effects on cancer cells; **C** Immunomodulation of microenvironment; **D** Metabolic reprogramming. MCT: Metronomic chemotherapy; TSP1: Thrombospondin 1; VEGF: Vascular endothelial cell growth factor; bFGF: basic fibroblast growth factor; HIF-1α: Hypoxia-inducible factor 1; EMT: Epithelial-mesenchymal transition; SA-β-Gal: Senescence-associated-beta-galactosidase; NK: Natural killer; OXPHOS: Oxidative phosphorylation.

The mechanisms of MCT were originally attributed to its antiangiogenic effects on dividing endothelial cells (Figure 2A) 46. Alternatively, conventional chemotherapy-induced cell death in endothelial cells could be circumvented by the secretion of endothelial cell survival factors, such as vascular endothelial growth factor (VEGF), basic fibroblast growth factor (bFGF), and angiopoietin 1 47. However, when chemotherapy is administered more frequently without extended breaks, as in MCT, the damaged endothelium has significantly fewer opportunities to undergo repair. This leads to the irreversible accumulation of antiangiogenic effects 27. Another study also demonstrated the antiangiogenic effects of metronomic 5-fluorouracil plus vinorelbine in triple-negative breast cancer (TNBC) through the disruption of FAK/VEGFR/VEGF signalling 48. In addition, several studies have indicated that thrombospondin 1 (TSP1) acts as a mediator of the effects of MCT and that its expression is closely correlated with antitumour effects 49, 50. Specifically, TSP1 primarily binds to CD36 receptors, which induces apoptosis of endothelial cells 51. The utilization of MCT consisting of low-dose cytotoxic drugs leads to fewer side effects, such as anaemia and myelosuppression 45, 52. This low-toxicity therapy offers the advantage of reducing the need for biopharmaceutical recombinant erythropoietin and recombinant methionyl granulocyte colony-stimulating factor (GCSF) compared with conventional chemotherapy 53. These cytokines typically promote the mobilization of marrow progenitor cells into the peripheral circulation, which may lead to worse outcomes 54. Furthermore, Bertolini *et al.* revealed that MCT has promising effects in preventing circulating endothelial progenitor (CEP) cell mobilization and inhibiting tumour growth, whereas MTD chemotherapy has been found to exert the opposite effects 55, 56.

In addition to the involvement of VEGF and the VEGF receptor family in tumour angiogenesis, the Notch signalling pathway is another key stimulator of vascular growth and tumour progression 57. As a receptor of the Notch signalling pathway, NOTCH-1 expression was previously reported to correlate with specific subtypes of breast cancer and chemoresistance 58, 59. Ilari *et al.* analysed the modulation of the expression of oncogenes and the cancer stemness-associated gene NOTCH-1 after metronomic therapy in patients with advanced TNBC 60. The immunoreactivity of NOTCH-1 shifted from prevalent at the time of diagnosis of metastatic TNBC to decreased membrane expression at relapse 60. As membranous NOTCH is cleaved, Notch intracellular domain (NICD) can translocate to the nucleus or remain in the cytoplasm to inhibit several oncogenic pathways, such as the c-MYC and AKT pathways 60.

Hypoxia, or low oxygen levels, is a common microenvironmental characteristic in many solid tumours 61. Hypoxia is closely linked to disease progression and poor survival rates due to increased potential for metastatic spread and resistance to cancer therapies. This challenging aspect of the TME contributes to the aggressiveness and treatment resistance of solid tumours, which indicates its importance as a target for novel therapeutic strategies, including MCT. The combination of low-dose chemotherapy, such as doxorubicin, with a limited number of antiangiogenic drugs can overcome resistance in tumours that exhibit enhanced HIF-1 activation, which leads to elevated VEGF-A levels and subsequent hypoxia 62. Consistently, metronomic cyclophosphamide has been found to offset HIF-1α induction and limit hypoxia in colon cancers 63. This approach effectively decreases HIF-1α, reduces VEGF expression, and inhibits angiogenesis, thereby rectifying the oxygen imbalance 64. These findings provide a compelling rationale for the combination of MCT and antiangiogenic drugs, as this treatment offers a potential synergistic approach to inhibit cancer growth and angiogenesis.

### Direct effects on cancer cells

As a novel schedule for chemotherapeutic agents, MCT affects cancer cells through various mechanisms, including the induction of cell death, direct reduction of cancer stem cells, inhibition of epithelial-mesenchymal transition (EMT), and promotion of senescence and subclone selection (Figure 2B) 43, 63. Numerous studies have indicated that MCT directly induces cancer cell death, including apoptosis and autophagy-dependent cell death 65-68. Compared with drugs administered alone, metronomic vinorelbine combined with Endostar exhibited significantly increased antitumour activity and induced apoptosis in Lewis lung carcinoma by downregulating Bcl-2 expression and upregulating the expression of Bax and caspase 3/7 69. Interestingly, Bruni *et al.* observed a shift from caspase-dependent to caspase-3-independent apoptosis when the dose of etoposide was decreased in the treatment of acute myeloid leukaemia (AML) 66. Similarly, another study revealed that metronomic temozolomide caused the accumulation of cytotoxicity through apoptosis, cellular senescence, and DNA damage to inhibit glioblastoma cells 70. In addition to its effect on apoptosis, MCT also exerts its antitumour effects through autophagy 71. The metronomic use of the podophyllotoxin derivative etoposide induced autophagy in non-Hodgkin's lymphoma through increased expression of Atg5, Beclin1, and LC3 71. Analysis of the neoadjuvant trial JBCRG-07 indicated that metronomic cyclophosphamide plus letrozole increased the expression of the autophagy-related markers Beclin 1 and LC3 in hormonal receptor (HR)-positive breast cancer tissues 67.

Vives *et al.* reported that metronomic cyclophosphamide could reduce the number of CD133^+^ precursor cells and triple-positive CD133^+^/CD44^+^/CD24^+^ cancer stem cells in orthotopic models of human pancreatic adenocarcinoma 72 (Figure 1). Similar targeting of drug-resistant CD44^+^ and CD133^+^ prostate cancer cells was also observed *in vivo* after treatment with a combined metronomic regimen of docetaxel and the metabolic blocker fenofibrate 73. Interestingly, in mice with CD44^+^CD49d^+^ lymphoma, only leukaemia cell apoptosis was observed after treatment with a combination of anti-CD44, anti-CD49d, and low-dose cisplatin, which disrupted apoptotic resistance to antibodies and drove apoptosis during chemotherapy 74 (Figure 1). These findings demonstrate that MCT can target CD44^+^ cancer stem cells and exert synergistic effects with CD44 blockade.

In addition, the metronomic application of several chemotherapeutics has been found to inhibit the EMT process in cancer cells 75, 76. Single-cell RNA-seq and RNA-seq analysis have indicated that metronomic topotecan treatment induced the downregulation of EMT markers, including CD55 and HAS3, in metastatic castration-resistant prostate cancer 76. A preclinical study revealed that metronomic cordycepin upregulated E-cadherin and downregulated N-cadherin protein expression in a human oral squamous cell carcinoma xenograft model, which suggests the EMT process was inhibited 75.

Senescent cells are characterized by cell cycle arrest, flattened cell bodies, and high levels of senescence biomarkers such as senescence-associated-beta-galactosidase (SA-β-Gal), p16 and p21 77, 78. Metronomic topotecan impedes tumour growth by inducing cell cycle arrest, p21WAF/CIP1 upregulation and DNA damage, but favourable NFKB1/p50 activation does not occur 79. Another study indicated that a significant increase in the number of senescent cells (characterized by SA-β-gal activity) was observed after metronomic 5-fluorouracil and vinorelbine was applied in TNBC 80.

Another mechanism by which MCT exerts direct effects on cancer cells is subclone selection. Tumours acquire more genetic mutations as they grow, which leads to greater heterogeneity during tumour progression 81. Consequently, the bulk tumour might include a diverse collection of subclones harbouring distinct molecular signatures with differential levels of treatment sensitivity. These subclones, comprising both sensitive and resistant variants, compete for space and resources within the TME. Phenotypic resistance to chemotherapy in cancer cells is attributed to the proliferation of chemoresistant cancer cells after chemosensitive cancer cells are eliminated 31, 82. Compared with MTD-based chemotherapy, MCT can restrain the proliferation of resistant subclones and hinder 'subclonal switching signals' to prevent dormant subclones from becoming the dominant subclones that can lead to tumour progression and recurrence 83, 84. In a transgenic mouse model of cancer, additional use of metronomic cyclophosphamide increased endothelial cell apoptosis and improved survival outcomes 83.

### Immunomodulation of the immune microenvironment

Accumulating evidence suggests that chemotherapeutic agents can modulate the immune microenvironment of tumours, which is crucial for the long-term control of cancer 85, 86. The immunological effects of chemotherapeutic drugs are extremely diverse due to variations in their mechanisms and dosing schedules 87. A key goal of MCT is to facilitate immunostimulation and promote the “cold to hot” transition of the microenvironment 88. Previous studies have indicated that MCT can promote the induction of immunogenic cell death (ICD) and increase the susceptibility of tumour cells to immune effectors 89, 90. ICD refers to a specific form of cancer cell death that is induced by certain chemotherapeutic drugs 91. This process is triggered by the release of damage-associated molecular patterns (DAMPs) from dying tumour cells, which leads to the activation of tumour-specific immune responses 91. Metronomic oxaliplatin was found to upregulate the expression of ICD markers, including calreticulin and high mobility group box-1 (HMGB1), both *in vivo* and *in vitro* 90. Consistently, metronomic cyclophosphamide was shown to trigger immunogenic cell death, as indicated by elevated calreticulin, adenosine triphosphate (ATP) release, and HMGB1 secretion 92.

Metronomic dosing with specific chemotherapies has shown certain desirable immune responses, such as the activation of cytotoxic T cells and natural killer (NK) cells, preferential depletion of regulatory T (Treg) cells, and enhancement of antigen presentation through the activation of dendritic cells (DCs) (Figure 2C) 22, 93-95. Despite numerous studies that have investigated the immunomodulatory effects of MCT, limited research has provided a comprehensive understanding of the precise impact of MCT in a high-resolution context 22. Recently, Bhavana and colleagues systemically described the dynamic changes in the immune microenvironment after metronomic doxorubicin and cyclophosphamide at the single-cell level and analysed the impact on myeloid cells 22 (Figure 1). They observed a highly heterogeneous transcriptome status of tumour-associated myeloid cells after MCT, which was indicative of a balance in the immune stimulatory and immunosuppressive environments in the TME. Specifically, chemotherapeutics were reported to induce a refreshed reconstitution of immune populations 96. Low-dose cyclophosphamide and paclitaxel have been shown to enhance NK cell effector functions and CD8^+^ T-cell activation in cancer patients 95, 97. Phenotypic and functional maturation of DCs could be induced by low-dose noncytotoxic vinblastine 33. Furthermore, MCT plays an important role in the elimination and inhibition of immunosuppressive cell populations 96. Previous studies have indicated that metronomic cyclophosphamide and gemcitabine suppress the infiltration and function of Tregs 95, 98.

In addition to the immunostimulatory function of MCT, some chemotherapeutic agents can increase the sensitivity of tumour cells to immune effectors such as cytotoxic T lymphocytes (CTLs) and NK cells 99, 100. Ramakrishnan *et al.* reported that common chemotherapeutic drugs could sensitize tumour cells to CTLs by increasing the permeability of tumour cells to granzyme B via the upregulation of mannose-6-phosphate receptors 99. Moreover, metronomic gemcitabine was found to promote the expression of MICA/B on the cell surface, which enhances innate immunity against tumour cells 101.

### Metabolic reprogramming

Metabolic reprogramming is regarded as a hallmark of carcinogenesis and cancer progression 102. However, growing evidence indicates that metabolic pathways and metabolites could function as regulators in signalling and in interactions and modulation between the immune microenvironment and cancer cells 103, 104. An integrated analysis of metabolomics and PCR array data revealed that MCT consisting of certain agents inhibits tumour growth via metabolic reprogramming in cancer cells, including the inhibition of glycolysis, amino acid metabolism and nucleotide synthesis-associated metabolic pathways (Figure 2D) 105. MCT has been shown to increase Hif-1a, Aldoa, and Pgk1 expression, which implies an upregulation of glycolysis in colon cancer 106. Moreover, in one study, metronomic doxorubicin significantly increased the concentrations of amino acids, especially alanine, aspartate, and glutamine, while the MTD of doxorubicin decreased the concentrations of these amino acids 105. Furthermore, metronomic doxorubicin slightly and broadly upregulated the expression of key enzymes involved in nucleotide metabolism, such as carbamoyl phosphate synthetase II, CTP synthetase 1, phosphoribosyl pyrophosphate aminotransferase, and IMP dehydrogenase 1 105.

OXPHOS activity was previously reported to correlate with the efficacy of chemotherapeutics 107. Oresta *et al.* indicated that the chemotherapeutic drug mitomycin C-induced ICD relies on the metabolic reprogramming of tumour cells towards increased oxidative phosphorylation (OXPHOS) activity 108. This process leads to increased mitochondrial permeability and the release of mitochondrial DNA into the cytoplasm, which subsequently activates the inflammasome to efficiently secrete interleukin-1β and promote DC maturation 108, 109. Consistently, metformin-induced disruptions in mitochondrial respiration were found to enhance the antitumour effects of metronomic cyclophosphamide in patients with neuroblastoma 107. Bondarenko *et al.* demonstrated that drug-sensitive NSCLC subclones mostly relied on aerobic glycolysis, while drug-resistant clones relied on OXPHOS 109. A similar finding was demonstrated in AML, in which chemotherapy-resistant AML cells exhibited increased mitochondrial mass and retained active polarized mitochondria, indicative of OXPHOS 110.

Furthermore, the anaerobic metabolism of cancer cells can lead to an increase in hydrogen generation, resulting in tumour acidity 111. The highly acidic microenvironment can quickly protonate and neutralize weak bases from chemotherapeutics, which triggers chemoresistance 112. However, patient alkalization through the use of proton-pump inhibitors (PPIs) or water alkalizers has been shown to be well tolerated and to enhance the tumour response to MCT agents 113.

## Application of MCT in oncology clinics

Since the inception of MCT in 2000, its clinical application has progressively expanded and has been accompanied by sustained growth in designed clinical trials. MCT has been applied to a range of cancer types, including breast cancer, NSCLC, gastrointestinal cancer, gynaecological cancer, and nasopharyngeal cancer 20, 114-117. Additionally, it has been utilized in cancers with comparatively lower incidence rates, such as oral cancer and nervous system tumours 118, 119. Therefore, the clinical application of MCT is extensive. MCT agents can not only be used in combination with various types of therapeutics, but this approach also offers low-toxicity and effective treatment regimens for elderly patients who are not suitable for intensive therapies 120.

MCT plays dual roles not only as a final salvage treatment for advanced tumours but also as a widely adopted maintenance therapy due to the convenience and minimal toxicity of the agents used 121. The primary agents used in MCT are mainly cyclophosphamide, methotrexate, vinorelbine, tegafur-uracil and capecitabine.

### MCT as salvage treatment

The predominant agents for single-agent metronomic therapy are capecitabine and vinorelbine. In the context of advanced breast cancer, a phase III clinical trial that compared the efficacy of capecitabine MCT (1,000 mg twice daily for 14 of 21 days) with classical cyclophosphamide, methotrexate, and fluorouracil (CMF) therapy as a first-line treatment confirmed the equivalence of single-agent metronomic therapy to capecitabine in terms of efficacy and also demonstrated superior tolerability 122. Notably, capecitabine monotherapy has yielded favourable outcomes even in patients with advanced disease. In a single-arm phase II clinical trial conducted by Fedele, late-stage breast cancer patients who received capecitabine monotherapy (1,500 mg once per day) achieved a clinical benefit rate (CBR) of 62%, along with a median time to treatment progression (mTTP) of 7 months 52. Consistently, metronomic capecitabine is a well-tolerated and effective therapeutic option for patients with liver cancer and those with gastrointestinal tumours 123-125.

Moreover, metronomic oral vinorelbine (mVNR) is regarded as a well-tolerated and effective therapeutic capable of achieving long-term disease control and stability in metastatic diseases 126, 127. When conventional chemotherapy is not feasible for elderly patients, the use of mVNR, which is less toxic, can serve as a rescue treatment, and an impressive CBR of 72% has been demonstrated in elderly patients with advanced NSCLC 128. Similarly, Addeo *et al.* observed good tolerability and promising results (with an objective response rate (ORR) of 38%) in a cohort of 34 patients with metastatic breast cancer (median age, 74 years) treated with the mVNR regimen (70 mg/m2, fractionated on days 1, 3, and 5, for 3 weeks on and 1 week off, every 4 weeks) 127.

The combination of cyclophosphamide and methotrexate (CM) constituted one of the earliest dual-drug metronomic regimens employed in oncology practice. In 2002, Colleoni *et al.* demonstrated that metronomic oral CM (cyclophosphamide 50 mg/day and methotrexate 2.5 mg twice per day on days 1 and 2 every week) was efficacious (with a CBR of 31.7%) and had low toxicity in the context of advanced breast cancer 129. Hussein *et al.* revealed the efficacy and decreased toxicity of metronomic oral CM regimens (cyclophosphamide 50 mg/day and methotrexate 2.5 mg twice per day on days 1 and 2 every week) in advanced breast cancer 130. While limited clinical studies have explored the application of metronomic CM in patients with advanced glioma (cyclophosphamide 100 mg daily and methotrexate 5 mg twice weekly) and prostate cancer (cyclophosphamide 50 mg/d and MTX 2.4 mg twice per week), the findings have not been encouraging 131, 132.

Additional dual-drug metronomic regimens, including cyclophosphamide with capecitabine (CX) and vinorelbine with capecitabine (NX), have demonstrated promising outcomes in clinical investigations. In a phase II study, CX (capecitabine 828 mg/m2 twice daily with cyclophosphamide 33 mg/m2 twice daily, days 1-14 every 3 weeks) was employed as a salvage treatment for human epidermal growth factor receptor 2 (HER2)-negative metastatic breast cancer, which resulted in a median progression-free survival (PFS) of 12.3 months and a CBR of 57.8% 133. The NX regimen has been established as an effective treatment for breast cancer, boasting a median time to progression (mTTP) of 10.5 months, an ORR of 33%, and a CBR of 67% 134.

In recent years, the three-drug combination metronomic regimen of vinorelbine and cyclophosphamide plus capecitabine (VEX) has achieved exceptional results in clinical trials. In a phase 2 clinical study, VEX (cyclophosphamide 50 mg daily, capecitabine 500 mg three times daily and vinorelbine 30 or 40 mg) achieved mTTP values of 25.1 and 11.2 months in untreated and treated breast cancer patients, respectively 135. In the subsequent randomized controlled study METEORA-II, VEX (cyclophosphamide 50 mg daily, capecitabine 500 mg three times daily and vinorelbine 40 three times per week) extended the time to treatment failure (TTF) by 2.5 months and the PFS by 4.2 months compared with weekly intravenous paclitaxel 17.

### MCT as a maintenance treatment

In addition to salvage treatment, MCT could be used as a maintenance treatment strategy, including maintenance following adjuvant chemotherapy or as maintenance therapy after salvage treatment (Table 1). Retrospective data have illustrated that adjuvant chemotherapy consisting of metronomic tegafur-uracil (2 capsules twice daily) could significantly improve the 5-year OS rate (71.6% vs. 28.7%, p<0.001) in patients with high-risk nasopharyngeal carcinoma after radiotherapy 136. In recent years, several high-impact, randomized, controlled, phase 3 trials have achieved impressive success with adjuvant treatment according to the MCT strategy 20, 21. Chen *et al.* conducted a phase 3 trial with 675 patients and reported that the addition of the metronomic adjuvant capecitabine (650 mg/m2 body surface area twice daily for 1 year) to chemoradiotherapy significantly improved failure-free survival (85.3% vs. 75.7%, p=0.002) in patients with high-risk locoregionally advanced nasopharyngeal carcinoma 20 (Figure 1). In another well-known trial with substantial cohorts, the SYSUCC001 study demonstrated that patients with early-stage triple-negative breast cancer could achieve a significant improvement in 5-year disease-free survival (DFS) from 1 year of treatment with metronomic capecitabine (650 mg/m2 twice a day) after adjuvant chemotherapy (82.8% vs. 73.0%, p=0.03) 21 (Figure 1). Moreover, maintenance chemotherapy with tegafur-uracil significantly enhanced both DFS and OS among patients with nasopharyngeal cancer 137. The option of first-line XELOX (500 mg bid daily) or FOLFOX (400 mg daily) followed by metronomic capecitabine maintenance is also considered for patients with colorectal cancer 138, 139.

## MCT combined with other therapies

### Combination with antiangiogenic agents

As mentioned above, MCT has been regarded as a drug scheduling scheme that is associated with lower toxicity and better tolerance, which makes it highly compatible for combination with other treatment modalities in clinical settings. Numerous ongoing clinical trials have aimed to determine the most effective synergistic therapies that can be combined with MCT to enhance antitumour effects and prolong the survival of cancer patients. Browder *et al.* demonstrated the potential effectiveness of combining MCT with endothelial cell-specific angiogenesis inhibitors, such as anti-VEGF receptor and COX-2 inhibitors, to achieve promising outcomes 33, 140.

**Figure 3:**
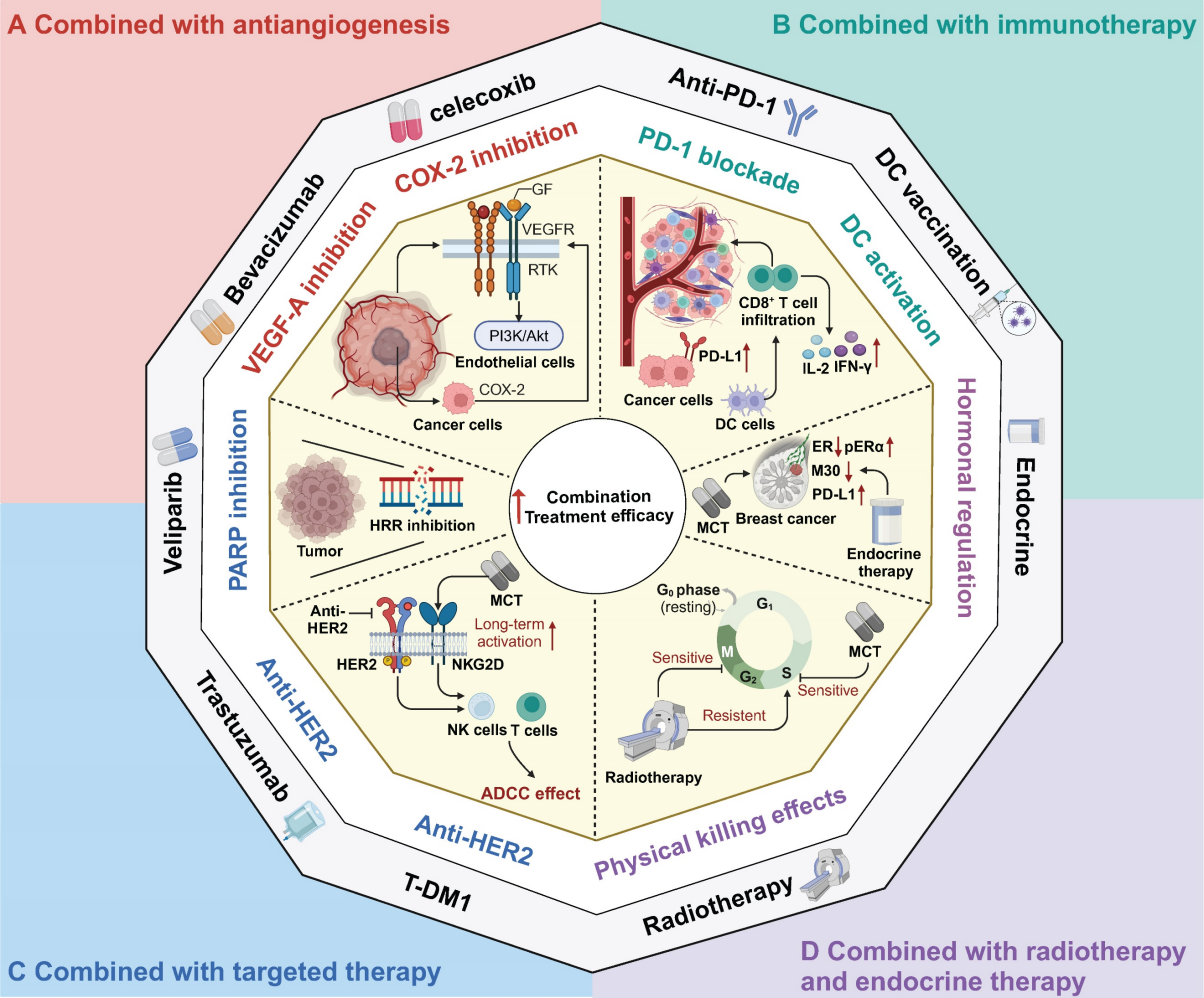
** Therapeutic strategies combined with metronomic chemotherapy in clinic. A** Combined with antiangiogenesis; **B** Combined with immunotherapy; **C** Combined with target therapy; **D** Combined with radiotherapy and endocrine therapy. MCT: Metronomic chemotherapy; GF: Growth factor; VEGF: Vascular endothelial cell growth factor; PARP: Poly(ADP‐ribose) polymerase; HRR: Homology-dependent recombination repair; PD-L1: Programmed Death Ligand 1; DC: Dendritic cells; IFN-γ: Interferon-gamma; IL-2: Interleukin 2; ER: Estrogen receptor; pERα: phosphorylated form of ER alpha; HER2: Human epidermal growth factor receptor 2; NK: Natural killer; ADCC: Antibody-dependent cellular cytotoxicity.

The rationale for the adoption of this strategy was based on several critical considerations (Figure 3A). Specifically, VEGF-receptor tyrosine kinases are preferentially expressed by endothelial cells within the actively growing neovasculature of a tumour, while COX-2 is expressed in both invasive and in situ cancer cells 141, 142. In one study, VEGF inhibited the apoptosis of endothelial cells within newly formed vessels by activating the PI3K-AKT pro-survival signalling pathway 143, 144. Furthermore, COX-2 has been found to upregulate the expression of the proangiogenic growth factor VEGF and to promote the inhibition of endothelial cell apoptosis through the stimulation of Bcl-2 or Akt activation 145.

In addition, it has been observed that conventional chemotherapy with docetaxel induces the expression of VEGF along with other antiapoptotic effectors 47. However, the elevated levels of VEGF promoted by conventional chemotherapy contribute to the development of multidrug resistance to chemotherapeutics 143, 146. Therefore, the combination of MCT with antiangiogenic drugs could enhance the proapoptotic effects of chemotherapeutic agents on proliferating endothelial cells. Numerous studies have demonstrated that VEGF inhibitors or COX-2 inhibitors combined with MCT may be associated with survival benefits in patients with various cancers 147-149. In a single-arm prospective clinical trial, the combination of the VEGF inhibitor bevacizumab and metronomic capecitabine and cyclophosphamide was found to be effective in advanced breast cancer 148. Similarly, a phase II trial demonstrated the effectiveness of bevacizumab combined with metronomic cyclophosphamide in patients with recurrent ovarian cancer 149. However, in a phase III randomized trial, patients with head and neck cancer who received the adjuvant metronomic methotrexate and celecoxib (a COX-2 inhibitor) failed to experience improvements in PFS or overall survival (OS) 147.

### Combination with immunotherapy

Due to its diverse range of immunomodulatory activities, MCT has emerged as an ideal candidate for combination therapy with immunotherapy, including drugs that function in immune checkpoint blockade (ICB) and immune vaccination 19, 150.

In recent years, ICB therapy has led to an antitumour immune response and has achieved great progress in various types of cancer, such as melanoma, NSCLC, and haematological malignancies 151-153. Despite its promising success in several cancer types, ICB therapy has not achieved satisfactory results in solid tumours such as breast cancer, colorectal cancer and hepatocellular carcinoma 154-156. Hence, to facilitate the transformation from a "cold" to a "hot" TME and to enhance the response rate to ICB, the combination of immunotherapy with conventional chemotherapy has been extensively explored and has yielded promising results, as demonstrated in trials such as Keynote-522, Keynote-048, and IMpower 130 6, 157, 158. Furthermore, the combination of MCT with immunotherapy has also shown synergistic effects in various types of cancer 159-161. MCT has immunostimulatory effects on both immune cells and cancer cells, which provides a rationale for the combination strategy of MCT and immunotherapy (Figure 3B). Khan and colleagues found that metronomic cyclophosphamide could increase the general immune response (and also upregulated PD-L1 expression) in preclinical models of breast cancer 159. Similarly, Zhou *et al.* confirmed this finding in their study on lung cancer, where they observed that low-dose carboplatin could facilitate the "cold-to-hot" transition of the TME, which resulted in increased infiltration of CD8^+^ T cells and increased PD-L1 expression 162. Furthermore, the combination of MCT with an anti-PD-1 agent demonstrated a substantial antitumour effect on squamous cell lung carcinoma 162. This effect was attributed to the increase in activated type I macrophages, DCs, and cytotoxic CD8^+^ T cells, as well as the preservation of intestinal gut microbiota diversity 162. In addition to ICB, MCT promoted the proliferation and infiltration of CD8^+^ T cells to sensitize patients with mesothelioma and metastatic melanoma to DC-based vaccines 163, 164.

In addition to the combination of chemotherapy and immunotherapy as a dual treatment approach, chemotherapy can also be synergistically combined with other treatment strategies to augment sensitivity to immunotherapy 22, 165. For instance, researchers have shown that MCT-induced treatment sensitizes TNBC to ICB treatment by chemically inhibiting STAT1 signalling 22. Similarly, in small cell lung cancer, the combination of metronomic gemcitabine with a checkpoint kinase 1 inhibitor has been found to enhance the efficacy of ICB therapy. This enhancement is achieved through activation of CD8^+^ cytotoxic T cells, DCs, and M1 macrophages, along with the downregulation of immunosuppressive M2 macrophages and myeloid-derived suppressor cells (MDSCs) 165.

Based on the rationale for the immunomodulatory effects of MCT, several clinical trials have further explored the combination of MCT with immunotherapy strategies in multiple cancer types 19, 117, 166. A phase III randomized trial demonstrated that the addition of low-dose nivolumab to MCT significantly increased the one-year OS from 16.3% to 43.4% 19. In another phase II study, Zsiros *et al.* reported that the combination of pembrolizumab with bevacizumab and metronomic cyclophosphamide achieved an impressive ORR of 47.5% and a median PFS of 10.0 months in patients with recurrent ovarian cancer 117. Moreover, a phase II trial revealed that when metronomic cyclophosphamide and a COX-2 inhibitor were given in conjunction with a DC vaccine, 57% of patients had achieved stable disease at the first evaluation 164. Another preclinical study demonstrated that MCT (taxanes and alkylating agents) plus a vaccine (a multipeptide cocktail including hepatitis C virus and tumour antigen TERT epitopes) enhanced the specific T-cell response and improved antitumour effects 167.

### Combination with targeted therapy

MCT combined with targeted therapy has shown considerable promise in multiple preclinical studies and clinical trials 168-171 (Figure 3C). Poly(ADP‐ribose) polymerase (PARP) inhibitors have been demonstrated to inhibit homologous recombination repair (HRR) and exhibit an immunomodulatory function 172. Kummar *et al.* conducted a phase I study, which provided evidence that the combination of veliparib and metronomic cyclophosphamide was well tolerated and exhibited encouraging activity in patients with BRCA mutations and refractory solid tumours and lymphoma 172. In another phase I study, this same combination treatment demonstrated consistent antitumour effectiveness in metastatic HER2-positive breast cancer 173.

Traditional anti-HER2 drugs, such as trastuzumab, primarily exert their antitumour effects through antibody-dependent cellular cytotoxicity (ADCC) 3, 174. Extensive research has demonstrated that trastuzumab can activate NK cells and T cells, enhancing the effects of ADCC and inhibiting HER2-positive breast cancer 175, 176. To further enhance therapeutic efficacy, chemotherapy can elevate interferon-gamma (IFN-γ) and interleukin 2 (IL-2) levels, which consequently activates cytotoxic T cells and NK cells 177. Currently, the combination of dual anti-HER2 targeted therapy with chemotherapy has become the prevailing approach in the neoadjuvant and adjuvant treatment of breast cancer 178, 179. However, limited attention has been given to exploring the combination of MCT and anti-HER2 targeted therapy. A preclinical study revealed that MCT combined with VEGF inhibition demonstrated antitumour effects on human breast cancer xenografts with acquired resistance to trastuzumab 180.

Orlando *et al.* conducted a phase II trial to show the clinical efficacy of the combination of metronomic cyclophosphamide and capecitabine with trastuzumab as a first-line therapy for HER2-positive breast cancer 181. Consistently, in a phase II randomized trial, the addition of metronomic oral cyclophosphamide to trastuzumab plus pertuzumab resulted in a significant increase in the median PFS of 7 months compared with dual HER2 blockade alone in patients with HER2-positive metastatic breast cancer 18. Additionally, a phase I trial of T-DM1 combined with metronomic temozolomide showed potential activity in the secondary prevention of HER2^+^ brain metastases 170.

### Radiotherapy combined with endocrine therapy

MCT regimens have also been investigated in combination with radiotherapy and endocrine therapy 182, 183 (Figure 3D). Chemoradiotherapy can be regarded as a form of "accelerated" radiotherapy, where radiation therapy is combined with anti-S phase radiosensitizing chemotherapy 184. Numerous studies have demonstrated that induction chemotherapy can improve the efficacy of radiotherapy in cancer patients 185-187. Furthermore, the combination of MCT and radiotherapy has shown potential for the development of treatment regimens with improved tolerability and increased response rates. For instance, a phase II trial evaluated the efficacy of metronomic vinorelbine combined with temozolomide and radiotherapy in breast cancer patients with previously untreated brain metastasis 188. Overall, 52% of patients (19/36) achieved either complete response or partial response after treatment with these combination regimens. In a recent clinical trial that evaluated the efficacy of bevacizumab, etoposide, and cisplatin in combination with whole-brain radiotherapy for previously untreated brain metastases in breast cancer patients, a comparative ORR of 52.6% was observed 189. Another retrospective study reported the PFS benefits of adding metronomic cyclophosphamide to radiotherapy versus radiotherapy alone in patients with NSCLC, although the response rates were not significantly different between these two groups 190.

Endocrine treatment with a selective oestrogen receptor modulator has been recommended as the first-line treatment for breast cancer patients who are positive for hormone receptors 191. In addition, endocrine therapy could be considered for patients with low-grade ovarian cancers and serous borderline ovarian tumours 192. MCT, when used in combination with endocrine therapy, has been extensively investigated in both preclinical studies and clinical trials 67, 193-195. Adamo *et al.* conducted a study to evaluate the effectiveness of oral metronomic vinorelbine in combination with endocrine therapy, specifically in HR-positive HER2-negative breast cancer 195. Another preclinical study indicated that the combination of metronomic 5-FU and exemestane exerted excellent tumour suppressive effects in gastric cancer 196.

Exploratory analysis of this combination strategy revealed an interesting observation: that this approach could induce an increase in PD-L1 expression and a decrease in oestrogen receptor (ER)-related gene expression 195. Furthermore, the phosphorylated form of ER alpha (pERα) was identified as an independent factor that affects the sensitivity of patients to letrozole plus metronomic cyclophosphamide therapy 197. Furthermore, in the clinical trial JBCRG-07, the addition of metronomic cyclophosphamide was shown to improve the therapeutic effects of letrozole in patients with HR-positive breast cancer 193. In response to metronomic chemoendocrine therapy, the levels of the apoptosis-related marker M30 decreased 67.

A multicentre phase II trial investigated the effectiveness of neoadjuvant letrozole plus low-dose cyclophosphamide in early-stage ER-positive breast cancer 193. This therapeutic combination demonstrated a favourable clinical response among the enrolled ER-positive breast cancer patients, with a clinical response rate of 67.5% 193. Similarly, in another randomized phase II trial, the combination of neoadjuvant letrozole and metronomic cyclophosphamide showed superior clinical efficacy compared with letrozole alone in early-stage breast cancer (disease response: 82.7% vs. 73.2%) 197. Beyond early-stage breast cancer, a phase II trial explored the use of metronomic capecitabine in combination with aromatase inhibitors in advanced breast cancer patients 198. This treatment regimen exhibited a significant ORR of 70.5% and achieved a PFS of 16.2 months when used to treat HR-positive advanced breast cancer patients who were treated with second-line therapy or beyond 198.

Health-economic benefits are derived from MCT. The progress achieved in cancer treatment in high-income countries is evident, as the average 5-year net survival in these countries is approximately 12 times greater than that in low-income countries 199. Despite that 75.1% of cancer-related deaths occur in low- and middle-income countries (LMICs), their proportion of the economic burden of cancer was comparatively lower at 49.5% 200. In addition, authors from the Harvard School of Public Health estimated that cancer treatment costs needed to increase the survival of patients with eleven cancer types will increase by 6.9% between 2020 and 2023 201. The disparities in medical resources and health outcomes and the increase in financial burdens underscore the need for oncologists and governments to prioritize the health-economic considerations of anticancer therapies. In the realm of cancer treatment options, MCT regimens are remarkable due to their cost-effectiveness and convenient administration 202. The reasons for this are that metronomic therapies are often associated with lower direct costs due to their inclusion of affordable generic drugs, many of which have been available for a long time 202. Moreover, the oral formulation of these metronomic drugs eliminates the requirement for expensive hospital stays, intravenous injections, and the use of central venous access, which results in additional cost savings 202. Finally, compared with MTD chemotherapy, MCT usually does not expose patients to a greater risk of infections or additional nutritional problems, which potentially decreases the need for monitoring, supportive care and fees for adverse effects 203.

For instance, several studies have compared the cost-effectiveness of MCT regimens to that of alternative chemotherapy regimens 204, 205. The use of metronomic capecitabine as an adjuvant chemotherapy has been shown to be a cost-effective treatment strategy in patients with locoregionally advanced nasopharyngeal carcinoma 204. In this cost-effectiveness analysis, the use of the adjuvant metronomic capecitabine resulted in an incremental cost-effective ratio (ICER) of $9,669.99 per quality-adjusted life year in China, which is significantly lower than the value of the WTP and even lower than the one-time per capita gross domestic product in China 204. Another comparative pharmacoeconomic assessment demonstrated that metronomic cyclophosphamide-methotrexate represents a notably cost-effective approach in the treatment of metastatic breast cancer 206.

In addition to improvement in survival rates, the assessment of treatment options and their influence on changes in health-related quality of life (HRQoL) holds significant importance, as it can provide valuable insights for health care practitioners. Several studies have investigated the correlation between the use of MCT regimens and their impact on HRQoL 207-211. Dal Lago *et al.* indicated that additional metronomic cyclophosphamide had no significant impact on HRQoL in older/frail patients with HER2-positive metastatic breast cancer 208. Moreover, metronomic methotrexate and celecoxib were found to significantly improve the pain QLQ-C30 score compared with cisplatin chemotherapy in patients with squamous cell carcinoma of the head and neck 211. Collectively, these findings substantiate the health-economic benefits of MCT regimens across a range of clinical contexts, especially in LMICs.

## Conclusion

After more than two decades since its inception, MCT has garnered increasing interest and remarkable success in cancer treatment. Recent discoveries of novel mechanisms across multiple dimensions of this approach, coupled with its combination with various therapies, have breathed new life into this traditional strategy. MCT should not be perceived merely as antagonistic; rather, MCT regimens can directly affect cancer cells, modulate the immune microenvironment, and induce metabolic reprogramming. Emphasizing the potential of combination approaches could expand the spectrum of MCT applications in clinical settings. Notably, when combined with antiangiogenic agents, immunotherapy, targeted therapy, endocrine therapy, and radiotherapy, MCT exerts promising synergistic effects against various cancers. In addition, compared with conventional chemotherapy, MCT offers the advantages of cost-effectiveness and reduced toxicity. Recently, the utilization of MCT has evolved from salvage treatment for metastatic disease to adjuvant maintenance therapy for high-risk cancer patients, which has been prompted by the success of several substantial phase III trials. The potential of MCT holds promise for its application in elderly and frail patients and in individuals with financial constraints.

## Figures and Tables

**Table 1:** Metronomic chemotherapy could be employed as maintenance treatment in various types of cancer.

Patient population/ disease setting	Type of study	N	Treatment regimen	Efficacy
Pretreated nasopharyngeal carcinoma	Retrospective	625	Tegafur-uracil	5-year OS: 71.6%
Triple negative breast cancer	Phase III	158	Cyclophosphamide with methotrexate	mPFS: 28m
Advanced ovarian carcinoma	Clinial trial	60	Cyclophosphamide with methotrexate	mPFS: 18m
Metastatic colorectal cancer	Clinial trial	233	Capecitabine	PFS: 66.7%
Colon cancer	Retrospective	132	Tegafur-uracil	5-year OS: 86.8%
Primary hepatic carcinoma	Clinial trial	114	Tegafur	PFS:16.25m; PFS%: 83.3%
Advanced oral cancer	Retrospective	356	Tegafur-uracil	5-year OS: 65%; 5-year DFS: 57%; 5-year DSS: 74%
Stage IV nasopharyngeal carcinoma	Retrospective	70	Tegafur-uracil	5-year OS: 91.89%
Metastatic colorectal cancer	Clinial trial	48	Capecitabine	mPFS: 5.66m; mOS: 23.82m
Locoregionally advanced nasopharyngeal carcinoma	Phase III	675	Capecitabine	3-year failure-free survival: 85.3%
Triple negative breast cancer	Retrospective	223	Cyclophosphamide with capecitabine / Cyclophosphamide with methotrexate	5-year DFS: 64.5%; 10-year DFS: 59.8%; 5-year OS: 71.2%; 10-year OS: 67.1%
Stage II colorectal cancer	Retrospective	233	Tegafur-uracil	5-year DFS: 81.39%
Locally advanced head and neck squamous cell carcinoma	Retrospective	240	Tegafur-Uracil	OS was not reached
Early-stage triple-negative breast cancer	Phase III	443	Capecitabine	5-year DFS: 82.8%; 5-year DSS:85.8%; 5-year OS: 85.5%
Pretreated nasopharyngeal carcinoma	Retrospective	98	Tegafur-uracil	mPFS:24.7m; mOS:36m

## Abbreviations

ADCC: Antibody-Dependent Cellular Cytotoxicity
AML: Acute Myeloid Leukemia
bFGF: basic Fibroblast Growth Factor
CBR: Clinical Benefit Rate
CEPs: Circulating Endothelial Progenitor Cells
CM: Cyclophosphamide and Methotrexate
CMF: Cyclophosphamide, Methotrexate, and Fluorouracil
CX: Cyclophosphamide with Capecitabine
CTLs: Cytotoxic T Lymphocytes
DC: Dendritic Cell
DFS: Disease-Free Survival
DSS: Disease Specific Survival
ER: Estrogen Receptor
GCSF: Granulocyte Colony-Stimulating Factor
GF: Growth Factor
HER2: Human Epidermal Growth Factor Receptor 2
HIF-1α: Hypoxia-Inducible Factor 1
HRQoL: Health-Related Quality of Life
HRR: Homology-Dependent Recombination Repair
ICB: Immune Checkpoint Blockers
ICD: Immunogenic Cell Death
IFN-γ: Interferon-Gamma
IL-2: Interleukin 2
mTTP: Median Time to Progression
MCT: Metronomic Chemotherapy
mOS: Median Overall Survival
mPFS: Median Progression-Free Survival
mVNR: Metronomic Vinorelbine
MDSCs: Myeloid-Derived Suppressor Cells
MTD: Maximum Tolerated Dose
NICD: Notch Intracellular Domain
NK: Natural Killer
NSCLC: Non-Small-Cell Lung Cancer
ORR: Objective Response Rate
OS: Overall Survival
OXPHOS: Oxidative Phosphorylation
PARP: Poly(ADP‐Ribose) Polymerase
PD-L1: Programmed Death Ligand 1
pERα: Phosphorylated Form of ER Alpha
PFS: Progression-Free Survival
PPI: Proton-Pump Inhibitors
SA-β-Gal: Senescence-associated-beta-galactosidase
Treg: Regulatory T Cells
TNBC: Triple-Negative Breast Cancer
TME: Tumour Microenvironment
TSP1: Thrombospondin 1
TTF: Time to Treatment Failure
VEX: Vinorelbine, Cyclophosphamide Plus Capecitabine
VEGF: Vascular Endothelial Cell Growth Factor
